# Systematic identification of characteristic chemical constituents in *Cistanches Herba* from saline-alkali land based on hierarchical filtering of mass spectrometry data and spatial metabolomics technology

**DOI:** 10.1186/s13020-026-01526-6

**Published:** 2026-10-19

**Authors:** Mengjiao Li, Yaqi Wang, Chenghao Bi, Ruifen Wang, Fengdan Guo, Guiqing Shen, Zihan Hu, Kairui Liu, Xinrui Li, Renwei Guan, Songsong Wang, Hao Zhong, Xingjun Wang, Liwen Han

**Affiliations:** 1 School of Pharmacy & Institute of Materia Medica, Shandong First Medical University & Shandong Academy of Medical Sciences, Jinan, 250117 Shandong China; 2 Shandong Academy of Agricultural Sciences Institute of Crop Germplasm Resources, Jinan, 250100 Shandong China; 3 Shandong University of Traditional Chinese Medicine, Jinan, 250014 Shandong China; 4 Shandong Xiandai University, Jinan, 250104 Shandong China; 5 Tianjin University of Traditional Chinese Medicine, Tianjin, 301617 China

**Keywords:** *Cistanches Herba*, Characteristic chemical markers saline-alkali land, Hierarchical filtering of mass spectrometry data, Spatial metabolomics

## Abstract

**Background:**

The traditional habitat of the Chinese medicinal plant *Cistanches Herba* (CH) is the deserts of northwestern China. Recently, CH cultivation has been introduced to saline-alkali land (SAL) in the Yellow River delta region. However, the effects of this SAL environment on the accumulation of secondary metabolites in CH remain unclear.

**Objective:**

This research aims to systematically characterize the chemical profiles of CH grown in SAL and to identify key chemical markers associated with the SAL environment.

**Methods:**

CH samples were collected from both SAL and traditional sandy land (SL). Untargeted metabolomics was performed using ultra performance liquid chromatography-quadrupole time-of-flight mass spectrometry (UPLC-QTOF-MS) to characterize the global metabolic profiles of CH from two habitats. Chemical markers associated with SAL environments were screened via weighted gene co-expression network analysis (WGCNA), targeted quantification by ultra performance liquid chromatography-triple quadrupole mass spectrometry (UPLC-TQ-MS), and compound loading contributions of principal component analysis (PCA). Subsequently, desorption electrospray ionization mass spectrometry imaging (DESI-MSI) was employed to visualize the in situ spatial distribution of partial screened constituents in mouse organs.

**Results:**

Untargeted UPLC-QTOF-MS metabolomics identified 74 chemical constituents from CH samples, including 40 phenylethanoid glycosides, 13 iridoid glycosides, 9 lignans, and other minor constituents. Orthogonal projections to latent structures-discriminant analysis (OPLS-DA) clearly separated the SAL and SL sample groups, with 30 constituents showing prominent contributions to the discrimination. WGCNA revealed 23 metabolites correlated with SAL. By integrating these two screening methods, 12 constituents were preliminarily selected as candidate markers associated with SAL. Subsequently, combining PCA loading coefficients (with the PCA model validated by Kaiser–Meyer–Olkin and Bartlett’s sphericity tests) and targeted UPLC-TQ-MS quantification, 4 constituents-verbascoside, echinacoside, isoacteoside, and pinoresinol- were ultimately identified as characteristic chemical markers, all exhibiting significantly higher levels in SAL samples. DESI-MSI results further demonstrated that echinacoside predominantly accumulated in brain tissue, whereas verbascoside or isoacteoside displayed the strongest ion signal in the lung.

**Conclusion:**

This study developed an integrated strategy that combines hierarchical filtering of mass spectrometry data to successfully identify 4 characteristic chemical markers of CH associated with SAL. This strategy provides a scientific basis for future quality control and resource development.

**Supplementary Information:**

The online version contains supplementary material available at https://doi.org/10.1186/s13020-026-01526-6.

## Introduction

*Cistanches Herba* (CH), a representative parasitic medicinal herb, is the scaly fleshy stem of the species *Cistanche deserticola* Y. C. Ma or *Cistanche tubulosa* (Schenk) Wight of Orobanchaceae family. *C. tubulosa* naturally parasitize the roots of the sand-fixing plant *Tamarix chinensis* Lour in the desert regions of China [1–3]. To improve saline-alkali land (SAL) in eastern coastal regions of China (e.g., the Yellow River delta), cultivating CH on the salt-tolerant *Tamarix chinensis* Lour has established a synergistic model that integrates ecological restoration with resource utilization, providing an effective land management strategy [4]. saline-alkali stress, a common abiotic stress factor, is known to influence the biosynthesis and accumulation of secondary metabolites in many medicinal plants, including increasing the levels of certain phenolic acids and flavonoids [5–7]. However, the systematic changes in the chemical composition of CH under saline-alkali conditions remains largely unexplored.

CH is a traditional Chinese medicinal herb used to invigorate kidney *Yang*, replenish essence and blood, and moisten constipation [8]. Modern research has shown that CH contains diverse chemical constituents, primarily including phenylethanoid glycosides, benzyl alcohol glycosides, iridoids, and lignans [9, 10]. Environmental stresses can modulate secondary metabolic fluxes, thereby altering the accumulation patterns of bioactive compounds. For example, saline-alkali stress significantly alters the secondary metabolite profiles of *Glycyrrhiza* and *Salvia miltiorrhiza* during later growth stages [11, 12]. Likewise, drought and salt stress induce marked changes in chlorogenic acid and luteolin levels in *Lonicera* [13, 14]. However, the characteristic chemical constituents of CH associated with saline-alkali stress remain poorly understood.

Liquid chromatography-high resolution mass spectrometry (LC-HRMS) has become a powerful tool for the identification, quality control, and pharmacological investigation of traditional Chinese medicine (TCM) [15, 16]. Untargeted metabolomics enables comprehensive profiling of metabolites, whereas targeted metabolomics allows precise quantification of specific constituents [17, 18]. In addition, spatial metabolomics techniques such as desorption electrospray ionization mass spectrometry imaging (DESI-MSI) allow visualization of the spatial distribution of constituents within tissues, providing direct evidence for elucidating the tissue targeting [19]. However, the diverse categories of MS techniques- covering quantitative, qualitative, and spatial distribution analyses-along with metabolomics approaches that employ various mathematical discrimination models, collectively generate massive datasets. Consequently, identifying key constituents of interest from the large datasets produced in TCM analysis remains a major challenge.

In summary, a hierarchical mass spectrometry screening strategy was established by integrating untargeted metabolomics, targeted metabolomics, multivariate statistical analysis, and weighted gene co-expression network analysis (WGCNA). Through this approach, 4 characteristic chemical markers of CH were successfully identified as being closely associated with the saline‑alkali habitat. The identified markers serve as reliable references for quality evaluation of CH cultivated in SAL, and further provide theoretical support for the quality control and sustainable exploitation of this medicinal material.

## Materials and methods

### Chemicals and reagents

The following chemicals and materials were used: verbascoside, echinacoside, verbasoside, cistanoside F, purpureaside C, pinoresinol, poliumoside, 8-epiloganic acid, sucrose, campneoside II, isoacteoside, and tubuloside A (purity ≥98%; Yuanye Biotech Co., Ltd., Shanghai, China) methanol, acetonitrile, and formic acid were purchased from Honeywell China Co., Ltd. (Charlotte, NC, USA). [Leu]-Enkephalin solution was acquired from Waters Corporation (Milford, MA, USA). Watson’s Distilled Water Co., Ltd. (Beijing, China) provided distilled water for the experiment. All additional chemicals and reagents were analytical grade.

### Plant materials

CH materials were collected from two standardized artificial cultivation areas: saline-alkali planting sites in Lijin County (Shandong Province, China) and desert planting sites covering Aral Township (Hotan, Xinjiang Uygur Autonomous Region), Fuhai County (Altay, Xinjiang Uygur Autonomous Region), Yutian County and Minfeng County (Xinjiang Uygur Autonomous Region, China).

In the Lijin planting site, the soil is sandy loam, the soil pH value is 8.32–8.68, the salt content is 1.75–2.07‰, and the exchangeable sodium content is 0.36–0.65, which belongs to mild SAL. From 2021 to 2024, the seeds of *C. tubulosa* were inoculated into the roots of one-year-old *Tamarix chinensis* Lour in April every year. In November, the sampling points with a spacing of ≥3–5 m were selected by random sampling method to collect the fleshy stem samples of *C. tubulosa*. A total of 10 batches of samples were collected from Lijin base and labeled as SAL-1 to SAL-10.

The desert sampling sites in Xinjiang are all long-term standardized cultivation bases that adopt cultivation protocols identical to those of the Lijin base: seeds were inoculated onto one-year-old *Tamarix chinensis* Lour seedlings every April, and all plant materials were harvested uniformly in November [20]. The soil in Xinjiang planting areas is aeolian sandy soil with a pH range of 7.4–7.83, and its total salt content is less than 0.7%, without accumulation of characteristic saline-alkali ions, which serves as the low-salt control habitat. A total of 10 batches of desert samples were collected and coded SL-1 to SL-10. All detailed information including exact sampling locations and harvest years for all 20 batches (10 SAL + 10 SL) is summarized in Supplementary Table S1.

All specimens were authenticated by Dr Fengdan Guo from Shandong Provincial Academy of Traditional Chinese Medicine as the fleshy stems with scale leaves of CH. Furthermore, DNA barcoding technology was applied for the molecular identification of CH samples. The internal transcribed spacer 2 (ITS2) region of nuclear ribosomal DNA was used as the barcode sequence for species authentication. After genomic DNA extraction and amplification of the target fragment, the ITS2 region was sequenced (Applied biosystems 3730xl DNA analyze). Subsequent NCBI BLAST results confirmed that all sequenced samples belonged to *Cistanche tubulosa* (the plant origin of *Cistanches Herba*). Detailed sequence alignment information is summarized in Supplementary Table S2. After cleaning, the fleshy stem tissues were cut into small pieces and stored at −80 °C for further processing.

### Animals

Male Kunming mice weighing (20 ± 2) g were purchased from Tai’an Hongfeier Biotechnology Co., Ltd. All animal experiments were reviewed and approved by the Institutional Animal Welfare and Ethics Committee of Shandong First Medical University (Approval No. 2023S6004). Throughout the study, mice were maintained in a clean-grade environment with a 12-h light–dark cycle, controlled temperature (20–24 °C) and humidity (50–60%), and free access to standard rodent chow and sterile water. The animals were randomly allocated into three groups (n = 7): a control group, a Shandong SAL of CH group, and a Xinjiang SL of CH group. SAL and SL groups received intragastric gavage of CH suspension at a dose of 5 g/kg (0.2 mL volume), while the control group was administered 0.2 mL volume of sterile distilled water. At 12 h after the final administration, mice were humanely euthanized via CO_2_ asphyxiation. Kidneys, heart, brain, and spleen were rapidly dissected, rinsed with normal saline, embedded in carboxymethyl cellulose (CMC), and stored at −80 °C for subsequent DESI-MSI analysis.

### Sample preparation

An appropriate amount of dried CH sample was ground into powder and passed through a No. 4 sieve for subsequent use. Precisely 0.25 g of each sample from different origins was weighed and transferred into a container. Then, 10 mL methanol (50%) was added, and the mixture was subjected to ultrasonic at a power of 250 W for 30 min. The mixture was centrifuged at 10,000 rpm for 10 min. The resulting supernatant was diluted to a final volume of 10 mL, yielding the sample extract for each CH specimen.

### Determination of soluble sugar, total phenolic, and total flavonoid contents

Soluble sugar content was quantified via the phenol–sulfuric acid method. 1.0 mL of sample solution was sequentially mixed with 0.5 mL of 6% phenol solution and 2.5 mL of concentrated sulfuric acid, vortexed immediately, and left to stand for 30 min, with absorbance measured at 490 nm using distilled water as the blank. Total phenolic content (TPC) was analyzed using the Folin–Ciocalteu assay [21]. Briefly, the supernatant was tenfold diluted, and 0.6 mL of the diluted solution was mixed with 0.2 mL of Folin–Ciocalteu reagent. After 5 min of reaction, 0.6 mL of 16% sodium carbonate solution was added, and the mixture was incubated in the dark for 30 min. The absorbance was then measured at 760 nm. Total flavonoid content (TFC) was determined by the sodium nitrite–aluminum nitrate method [22]. Briefly, 3 mL of sample solution was diluted to 6 mL with 30% ethanol, reacted sequentially with 0.3 mL of 5% sodium nitrite (5 min) and 0.3 mL of 10% aluminum nitrate (6 min), then treated with 2 mL of 1.0 mol/L sodium hydroxide, diluted to 10 mL with 30% ethanol, and allowed to stand for 15 min before absorbance measurement at 510 nm. Results were calculated using the standard curve equation Y = 0.6299X + 0.0326 and expressed as rutin equivalents (mg RE/g DW).

### Untargeted metabolomics analysis

CH powder (0.25 g) was soaked in 10 mL with 50% methanolfor 30 min and extracted by ultrasonication at 250 W and 35 kHz for 40 min. After centrifugation at 10,000 r/min for 10 min, the supernatant was filtered through a 0.22 μm membrane to obtain test solutions. Individual standard stock solutions of the nine constituents were prepared in methanol at a concentration of 1 mg/mL and further diluted to 10 μg/mL as working solutions. Analyses were carried out on a Waters SYNAPT XS TOF system with an ACQUITY BEH C18 column (1.7 μm, 2.1 × 100 mm) at 40 °C, using 0.1% formic acid aqueous solution and acetonitrile as mobile phases for gradient elution. The flow rate was 300 μL/min, autosampler temperature 4 °C and injection volume 2 μL.

Mass spectrometry was performed in negative ESI mode with optimized ion source parameters. Consistent with extensive published literatures focusing on CH chemical profiling, negative ESI mode delivers superior ion response and abundant diagnostic fragment ions for phenylethanoid glycosides, lignans and iridoid acids [23, 24]. Therefore, only negative ion mode data were subjected to metabolite annotation, multivariate statistical analysis and figure plotting, while positive ion mode data were excluded from formal analysis due to insufficient data quality and incomplete metabolic coverage. Spectra were acquired within m/z 50–1000 Da using leucine enkephalin as lock mass, and data were collected at two collision energy levels. Raw data were processed by MassLynx and UNIFI software. For differential-metabolite screening based on OPLS-DA, metabolites satisfying VIP > 1, *P* < 0.05 together with fold-change ≥1.5 or ≤0.5 were preliminarily regarded as differential candidates. Weighted gene co-expression network analysis (WGCNA) was applied to construct metabolite co-expression modules, and modules exhibiting high correlation with saline-alkali or sandy-land habitat phenotypes were extracted (RStudio). Candidate metabolites from phenotype-associated modules were further subjected to UPLC-TQ-MS-MRM absolute-quantitative filtering in targeted metabolomics analysis to screen stable habitat-related characteristic constituents.

### Targeted metabolomics analysis

Reference standards were dissolved in 50% methanol–water to prepare 1 mg/mL stock solutions, which were mixed and diluted to 50 μg/mL mixed standards and further serially diluted for subsequent tests; all standard solutions were stored at −20 °C. Sample preparation followed the same extraction procedure as described above, and the final extract was diluted tenfold, filtered and preserved at −20 °C. Targeted quantification was performed on an AB SCIEX Triple Quad 5500 coupled with an Exion LC system in MRM negative mode, with relevant ion parameters set as specified, while data acquisition and processing were completed by Analyst 1.6.3 and Sciex OS 1.4.0. Methodological validation was conducted by evaluating linearity using a series of mixed standard solutions, solution stability within 24 h, instrumental precision and accuracy via repeated injection, method repeatability with six parallel samples, and spike recovery tests.

### DESI-MSI analysis

After euthanasia of mice via CO_2_ inhalation, five organs including brain, heart, spleen, lung and kidney were rapidly dissected. The isolated tissues were rinsed with pre-cooled 0.9% normal saline to wipe off residual surface blood. Each tissue was fully embedded with CMC embedding medium and snap-frozen in an ultra-low temperature freezer at −80 °C for no less than 2 h. Tissue blocks were mounted on a Leica CM1950 cryostat maintained at −20 °C, and serial coronal sections with a uniform thickness of 50 μm were prepared [25]. The sections were mounted onto Epredia Superfrost® Plus positively charged adhesive glass slides, followed by vacuum drying at room temperature for 30 min to remove free water. The slides were immediately subjected to DESI-MSI analysis.

A stock calibration solution was prepared by dissolving (3.0 ± 0.15) mg of [Glu^1^]-fibrinopeptide B powder in 7.5 mL of ultrapure water. The electrospray solution was prepared fresh prior to analysis by adding the working calibration solution to a methanol–water mixture to achieve a final concentration of 200 ng mL⁻^1^. Imaging data acquisition, processing, and visualization were performed using High Definition Imaging (HDI) 1.5 software (Waters Corporation, Manchester, UK). Mass spectrometric data were acquired and processed with MassLynx version 4.2 (Waters Corporation, Manchester, UK). Ion intensity values for each tissue slice were obtained from centroid mass spectra exported from HDI software. The average intensity per unit area (mm^2^) of each lipid ion was calculated to compare its relative abundance across the eight tissues.

### Statistical analysis

Statistical analyses and data visualization were performed using GraphPad Prism 10.0 software. One-way analysis of variance (ANOVA) was used for statistical analysis of the data. Differences with *P* < 0.05 were considered statistically significant, while *P* < 0.01 and *P* < 0.0001 indicated extremely significant and highly significant differences, respectively.

## Results

### Botanical and chemical features of CH differ between SAL and SL habitats

**Fig. 1:**
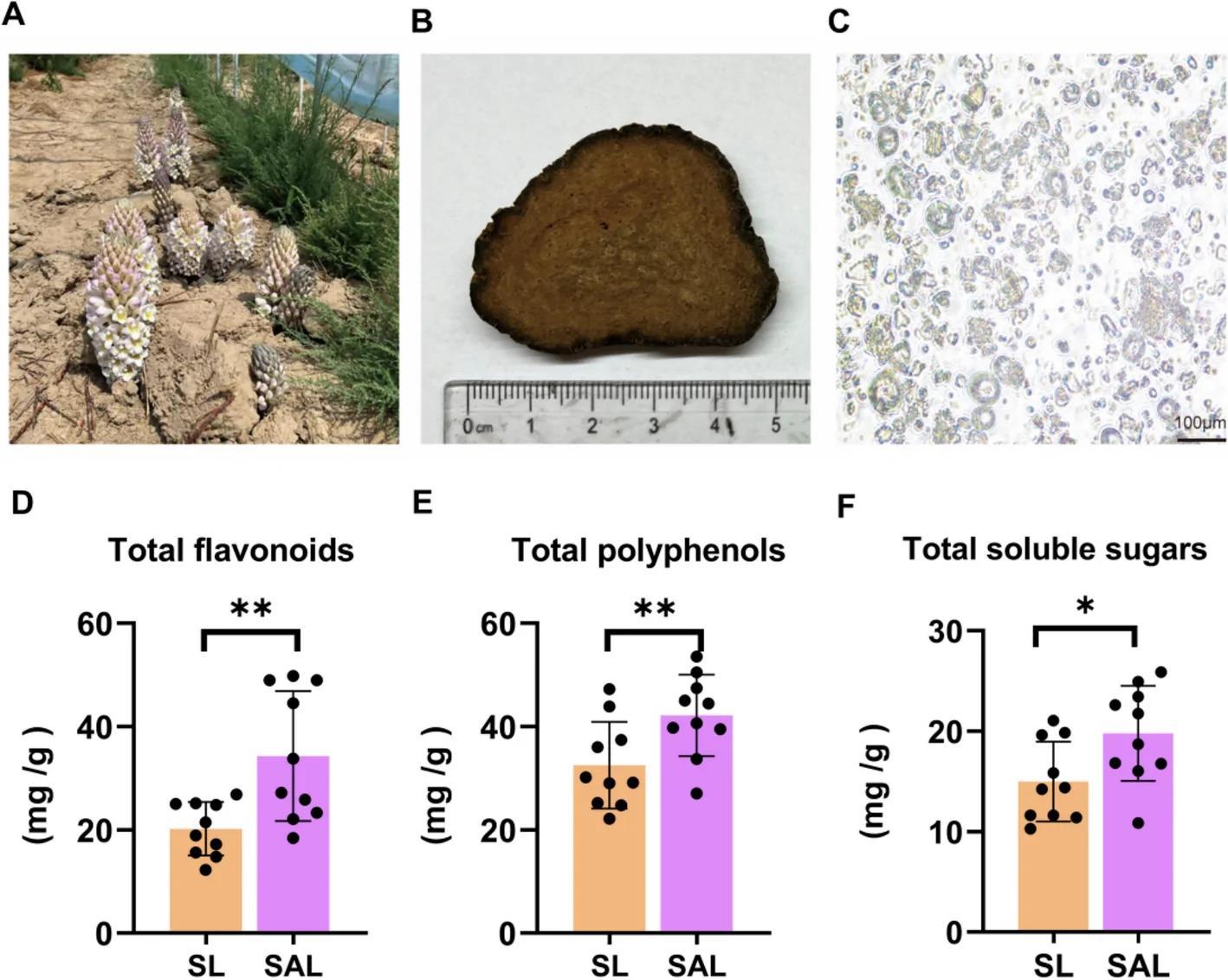
Botanical characteristics and comparison of major bioactive constituents profiles of CH from SAL and SL habitats. **A** Plant morphology of CH from SAL. **B** Characteristics of the sliced crude drug. **C** Starch granule features observed under an optical microscope. **D**–**F** Comparison of the contents of total flavonoids, total polyphenols, and soluble sugars in CH from SAL and SL. *P < 0.05, **P < 0.01

The morphological characteristics of *Cistanches Herba* (CH) samples collected from saline-alkali land (SAL) (Fig. 1A, B) were examined. The medicinal material was observed to be cylindrical or flattened-cylindrical in shape, slightly curved or bent. The surface was found to be yellowish-brown to dark brown, and the texture was hard and tough. The cross-section was yellowish-brown to dark brown, with numerous yellowish-white punctate vascular bundles arranged in an imbricate pattern. The surface was densely covered with fleshy scales, which were relatively thin and ovate to ovate-lanceolate. When the material was ground, a yellowish-brown powder was obtained. Starch granule was observed under an optical microscope (Fig. 1C), the starch grains were observed to be predominantly single, with a few compound grains consisting of two subgrains; the overall grain morphology was small.

A quantitative analysis of key stress-related compounds from CH cultivated in SAL was conducted and compared with samples from traditional sandy land (SL). The total polyphenol content in from CH cultivated in SAL was determined to be 42.16 ± 7.46 mg/g, the total flavonoid content was 34.29 ± 11.92 mg/g, and the soluble sugar content was 19.78 ± 4.49 mg/g, which was significantly higher than that measured in samples from SL origins (Fig. 1D-F).

### Identification of chemical constituents from CH by UPLC-Q/TOF–MS

**Fig. 2:**
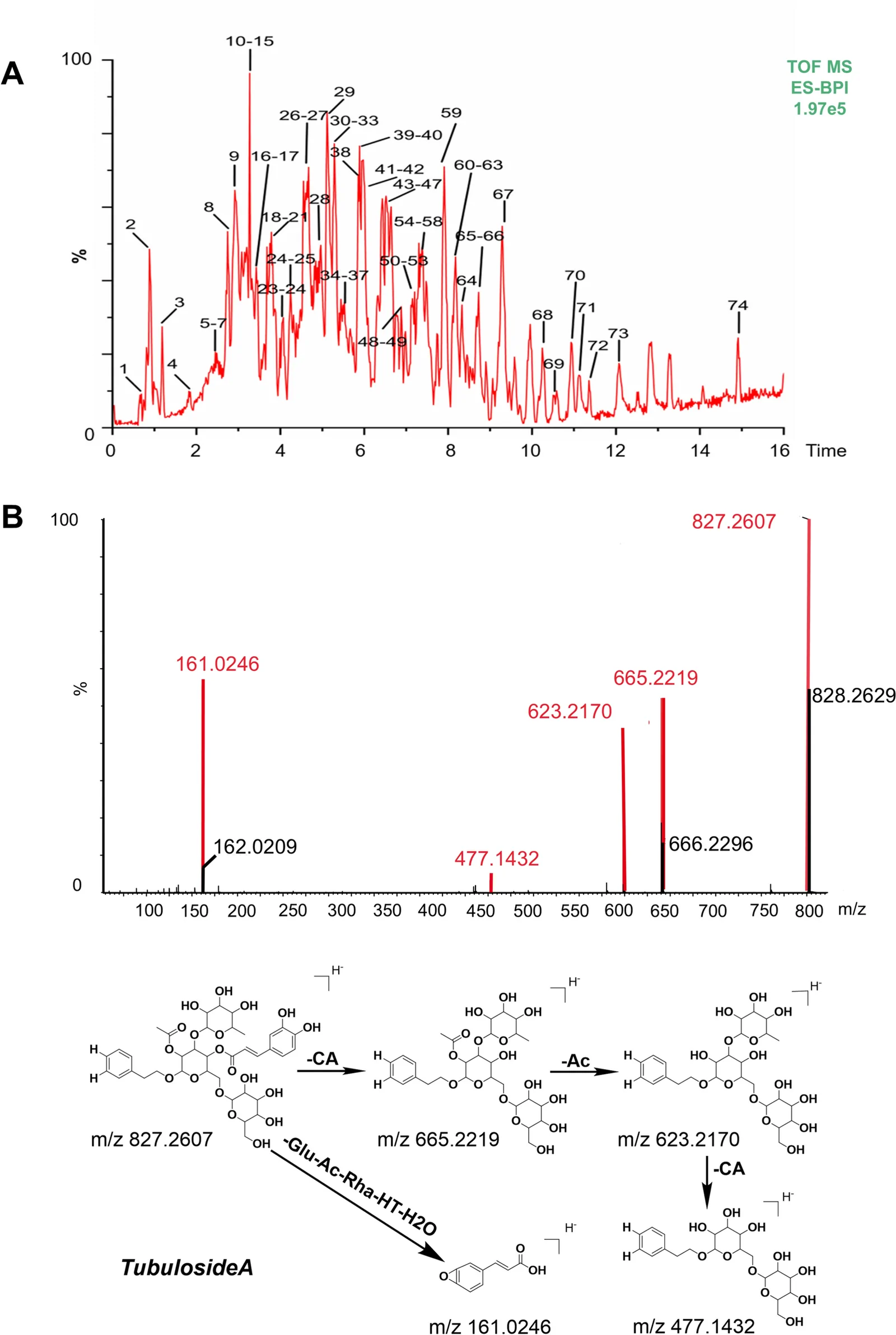
Chemical profiling and constituent identification of CH. **A** Total ion chromatogram of CH. **B** Fragmentation pattern of representative phenylethanoid glycosides

A total of 74 constituents were identified, including 40 phenylethanoid glycosides, 13 iridoid glycosides, 9 lignans, and 12 constituents belonging to other classes. Detailed information regarding these constituents including the names, retention times, and molecular formulas is provided in Table S3. Phenylethanoid glycosides constitute the primary chemical constituents of CH. Their chemical structures are characterized by a β-glucopyranose core, with the aglycone typically being a hydroxyphenylethyl moiety. Analysis of the MS/MS spectra and fragmentation patterns of phenylethanoid glycosides revealed consistent mass spectrometric fragmentation pathways. In negative ion mode, the major cleavage routes include: (1) Ester bond cleavage: loss of a neutral caffeoyl moiety (C_9_H_8_O_4_, 179.03 Da) or its dehydrated product (C_9_H_6_O_3_, 161.02 Da), and loss of a neutral acetyl group (C_2_H_2_O, 42.00 Da); (2) Glycosidic bond cleavage: loss of a neutral rhamnosyl residue (C_6_H_10_O_4_, 146.05 Da) and a neutral glucosyl residue (C_6_H_10_O_5_, 162.05 Da). Taking constituent 25 as an example, its quasi-molecular ion peak was observed at m/z 639.1940 [M–H]⁻. Loss of a water molecule yielded a fragment ion at m/z 621.1882 [M–H–H_2_O]⁻, which subsequently lost a dehydrated caffeoyl (CA) group to produce the fragment ion at m/z 459.1509 [M–H–H_2_O–CA]⁻. The fragment ions at m/z 179.0248 and m/z 161.0248 corresponded to [CA–H]⁻ and [CA–H_2_O]⁻, respectively. By comparison with literature data, this constituent was tentatively identified as the phenylethanoid glycoside campneoside II (Fig.2). The fragmentation patterns of the remaining constituents are detailed in the supplementary material (Figs. S1–S3).

### Metabolomics-based identification of differential constituents from CH

**Fig. 3:**
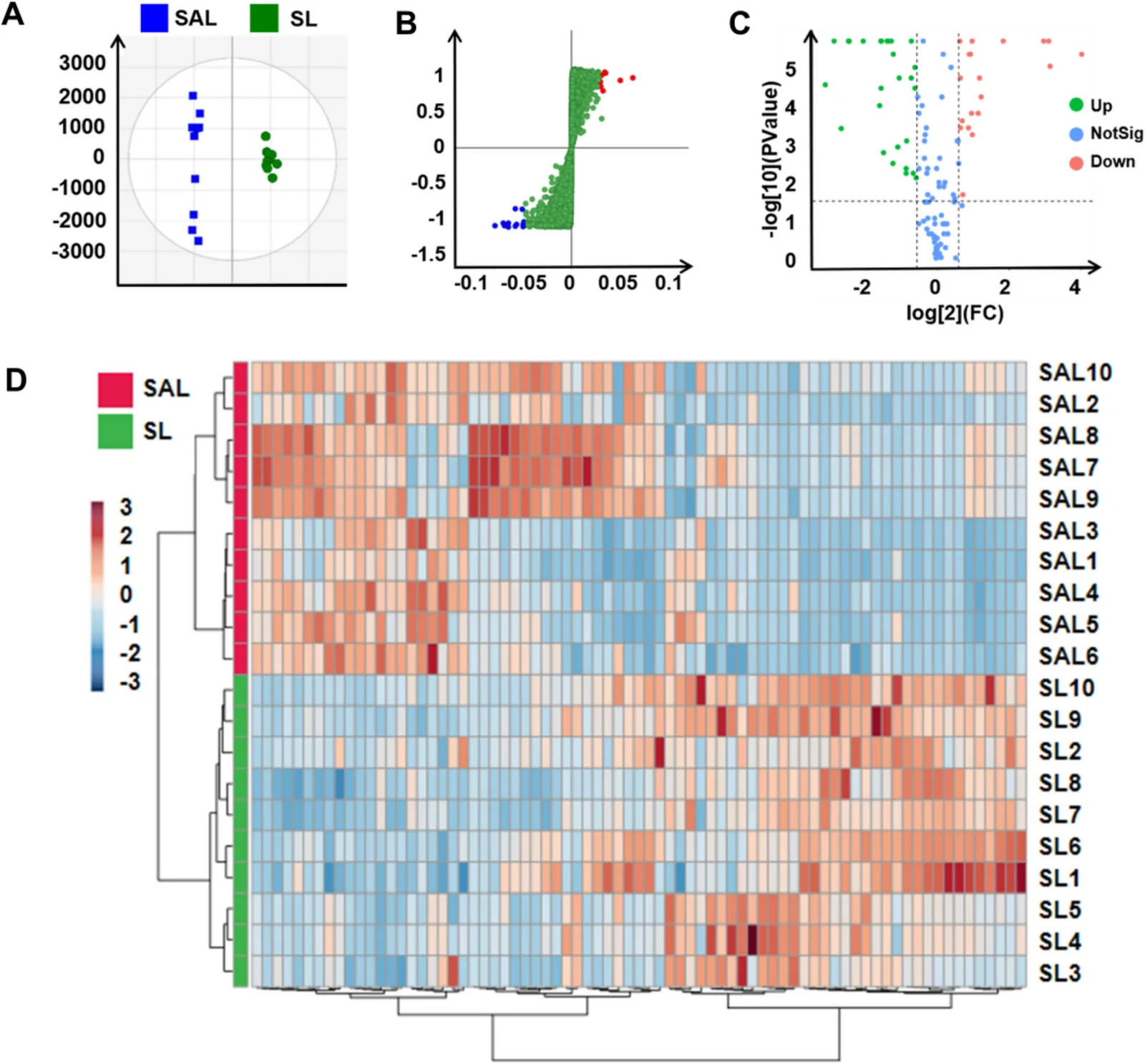
Identification of potential differential chemical constituents of CH between SAL and SL based on metabolomics technology. **A** OPLS-DA score plot. **B** Permutation test. **C** S-plot. **D** Volcano plot analysis

To identify potential differential chemical constituents in CH from SAL, metabolomics technology was employed to profile the overall chemical composition of samples from both SAL and SL. The result of Principal component analysis (PCA) revealed a clear separation trend between CH samples from the two habitats, indicating significant differences in their chemical profiles (Fig. S4A). Furthermore, orthogonal partial least squares discriminant analysis (OPLS-DA) model achieved R2Y and Q2(cum) values of 0.835 and 0.787, respectively (Fig. 3A). A permutation test (n = 200) was conducted to validate the model. All permuted R2 and Q2 values were lower than the original points on the right, and the intercept of the Q2 regression line was negative, confirming that the model was not overfitted (Fig. S4B).

S-Plot (Fig. 3B) and volcano plot analyses were then utilized to pinpoint the metabolites contributing most significantly to the group separation. The volcano plot analysis (Fig. 3C) indicated that 42 metabolite features exhibited significant changes. Compared with SL samples, 24 constituents increased and 18 constituents decreased in CH collected from SAL. Using the thresholds of VIP > 1, *P* < 0.05 and fold change (FC) ≥1.5 or ≤0.5, 30 potential differential metabolites were screened out between the two groups. As illustrated in Fig. 3D, the chemical composition of SAL and SL samples displayed distinct differences in the color gradient of the heatmap, further corroborating the findings from the PCA.

### WGCNA-based screening of candidate markers from CH cultivated in SAL

**Fig. 4:**
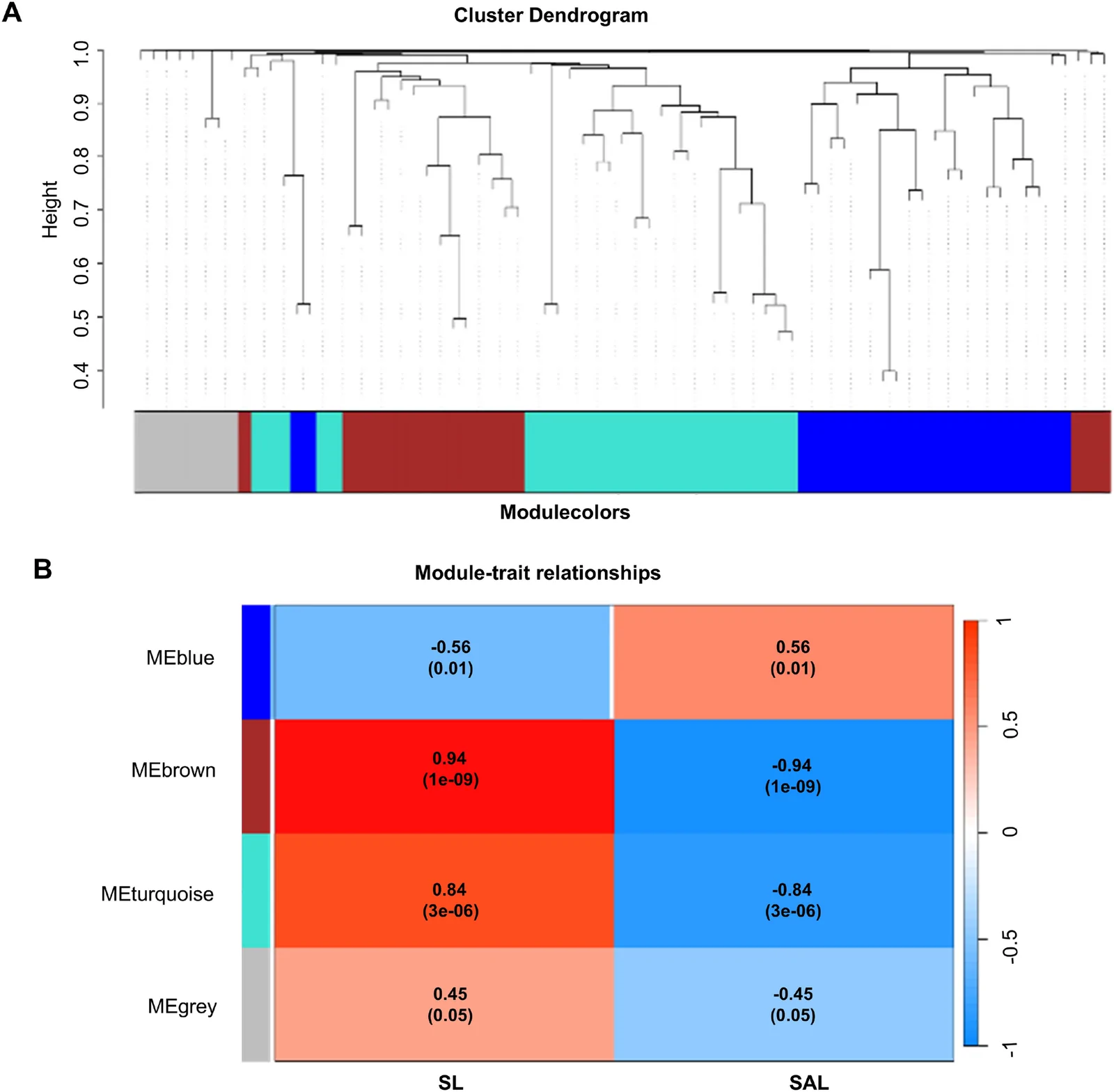
Screening of candidate biomarkers based on WGCNA analysis. **A** Metabolite co-expression module dendrogram. **B** Module-sample trait relationship heatmap

WGCNA is a widely used approach for identifying sets of genes or metabolites exhibiting highly coordinated changes. The 74 chemical constituents were re-analyzed using the WGCNA method to investigate the relationship between chemical composition and habitat conditions. Four co-expression modules were ultimately obtained, and metabolites not assigned to any modules colored in grey (Fig. 4A). The identified modules comprised the blue module (23 constituents), turquoise module (26 constituents), and brown module (18 constituents); the grey module was excluded from further analysis. Pearson’s correlation coefficients were calculated to evaluate the relationship between each module and the two sample groups (Fig. 4B). The blue module showed a significant positive correlation with the SAL group (r = 0.56, *P* < 0.05).

Integrated analysis with plant metabolomics revealed that 12 chemical constituents within the blue module overlapped with the putative differential metabolites of CH from SAL. As shown in Table S4, these included 9 phenylethanoid glycosides, 1 iridoid glycoside, 1 lignan, and 1 constituent from other chemical classes.

### Targeted metabolomics-based quantification of the constituents in CH

The precise contents of 12 constituents were determined using liquid chromatography–tandem mass spectrometry (LC–MS/MS). This quantitative analysis served as the basis for further screening of characteristic chemical markers specific to CH from SAL. In negative ion mode, all target analytes demonstrated correlation coefficients (R^2^) between 0.9989 and 0.9997 (Fig. S5), indicating that the established multiple reaction monitoring method was highly reliable of quantifying these constituents and meet the requirements for sample analysis.

Methodological validation demonstrated good stability: the relative standard deviations (RSDs) for the contents of all target constituents (n = 6) were below 2% after storage of the sample solution at room temperature for 24 h (Table S5). The precision results, as shown in Table S6, yielded RSDs between 0.47 and 2.21%, with relative error (RE%) ranged from 98.08 to 103.78%, confirming satisfactory precision and accuracy. Repeatability was assessed using six independently prepared test solutions, giving RSDs of 0.79–2.25% for each constituent (Table S7). Extraction recoveries ranged from 97.39 to 104.40%, with RSDs of 0.36–2.53%, indicating acceptable recovery performance (Table S7).

**Fig. 5:**
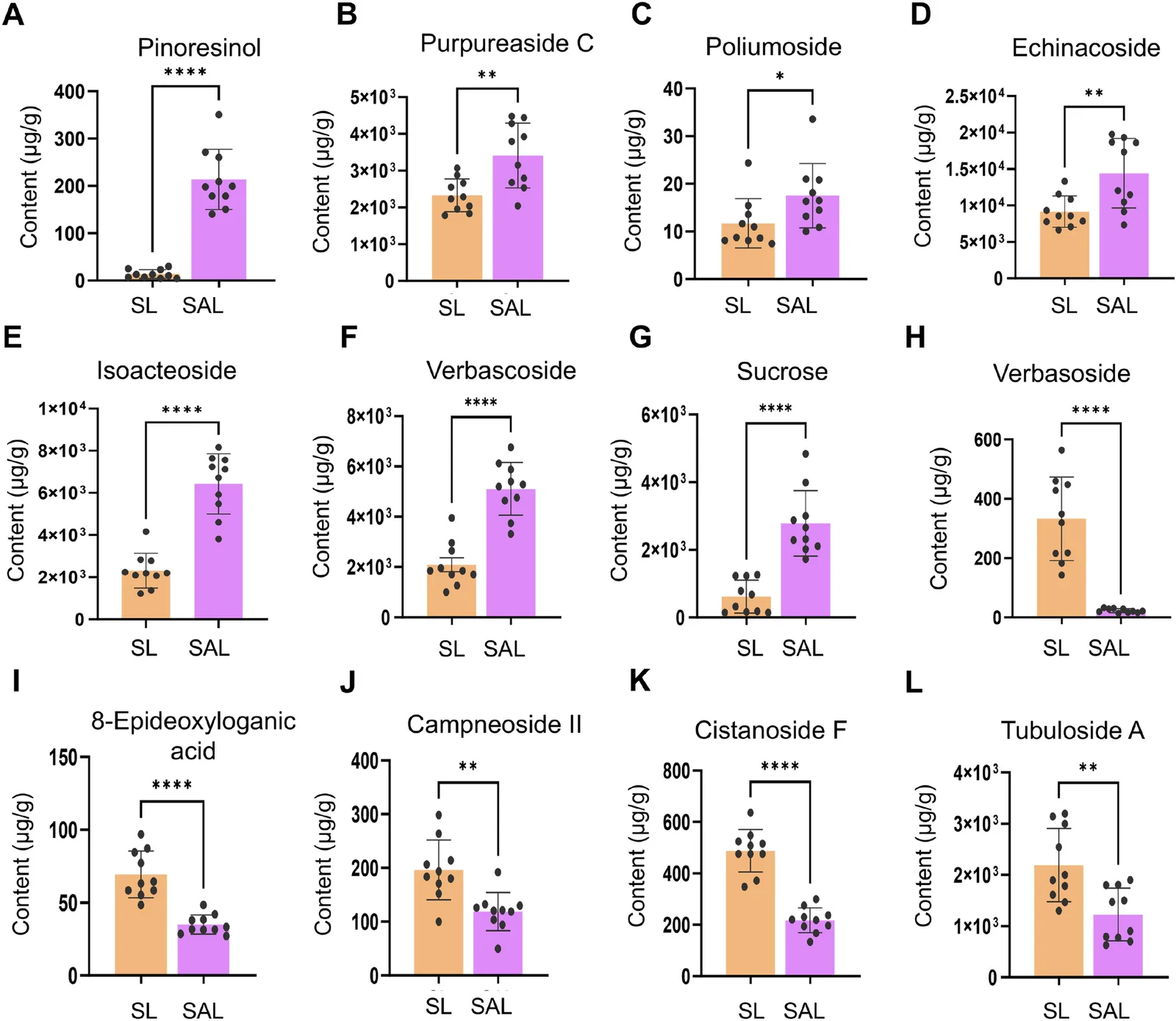
Quantitative comparative analysis of 12 characteristic constituents of CH from SAL and SL based on targeted metabolomics. Values are expressed as the mean ± SEM; *P < 0.05, **P < 0.01, ***P < 0.001

The quantitative results for the 12 constituents in CH from SAL and SL are shown in Fig. 5, and their chemical structures are shown in Fig. S6. Compared to the SL samples, the contents of 7 constituents were elevated in the SAL samples. Among these, isoacteoside showed the most pronounced increase, with an average content of 2313.60 ± 745.76 µg/g in desert-origin samples versus 6425.60 ± 1289.63 µg/g in SAL samples.

### Validation of characteristic chemical markers in CH associated with SAL

**Fig. 6:**
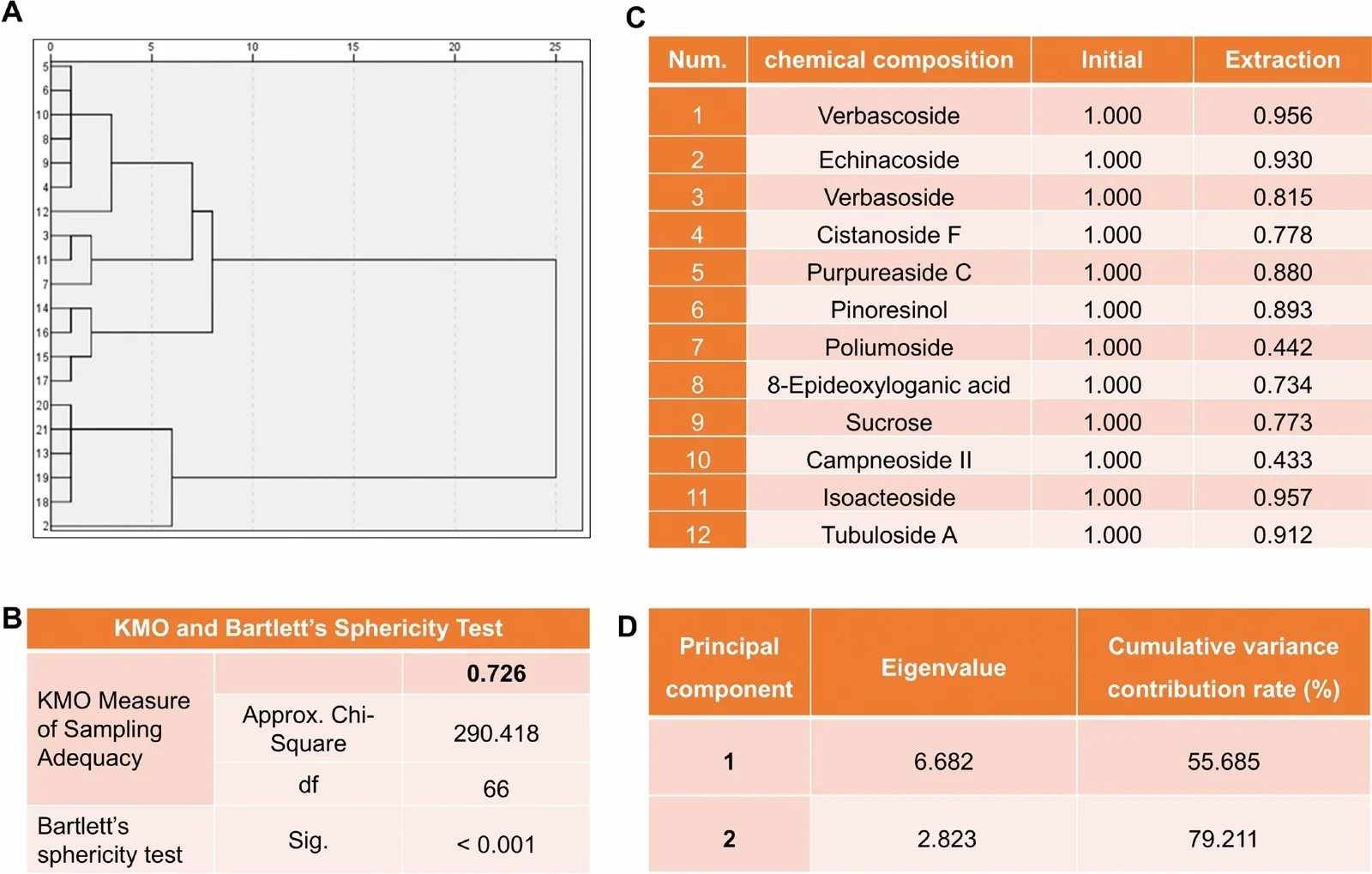
Multivariate pre-analysis of the dataset comprising twelve target constituents of CH. **A** Cluster analysis of quantitative results. **B** Results of KMO measure and Bartlett’s sphericity test. **C** Communalities of twelve constituents. **D** Scree-plot for principal-component selection

Cluster analysis (Fig. 6A) was performed using the 12 measured constituents as variables. Most CH samples from SAL fell into one group, whereas samples from SL clustered into the other group. This result revealed geographically dependent differences in compound contents, without obvious divergence at the species level. The Kaiser–Meyer–Olkin (KMO) measure and Bartlett’s sphericity test were employed to assess the suitability of the data for PCA.

The KMO value was calculated as 0.726. According to Kaiser’s criteria, this value verified that the variables were suitable for factor analysis. Bartlett’s sphericity test produced a statistical value of 290.418 with *P* = 0, demonstrating a significant difference between the correlation matrix and the identity matrix (Fig. 6B). Communality analysis showed that the communality values for most of the 12 constituents exceeded 0.7 (Fig. 6C), suggesting that most variables could be well explained by the extracted common factors. Based on the eigenvalue-greater-than-1 criterion, two principal components were extracted from the 12 chemical constituents, with a cumulative variance contribution rate of 79.211% (Fig. 6D).

In PCA, the loading coefficients reflect the degree of correlation between each original variable and the extracted principal components. Isoacteoside, verbascoside, pinoresinol, and cistanoside F exhibited high loadings on the first principal component, whereas tubuloside A and echinacoside showed high loadings on the second principal component (Table 1). An examination of the contents of these six constituents in CH revealed that four of them-verbascoside, echinacoside, isoacteoside, and pinoresinol-were significantly more abundant in the SAL samples than those from the SL habitat. Accordingly, these four constituents were selected as the characteristic chemical markers for discriminating CH derived from SAL. Finally, verbascoside, echinacoside, isoacteoside, and pinoresinol were confirmed as the final characteristic markers.

**Table 1:** Loading coefficients of 12 potential characteristic constituents from SAL-cultivated CH

Chemical constituents	Component score coefficient	
	Principal component 1	Principal component 2
Verbascoside	0.955	0.209
Echinacoside	0.596	0.758
Verbasoside	−0.771	0.470
Cistanoside F	−0.860	0.197
Purpureaside C	0.776	0.527
Pinoresinol	0.936	−0.130
Poliumoside	0.523	0.411
8-Epideoxyloganic acid	−0.751	0.413
Sucrose	0.742	−0.471
Campneoside II	0.520	−0.404
Isoacteoside	0.966	0.154
Tubuloside A	0.104	0.950

### Evaluation of spray solvent composition for DESI-MSI

**Fig. 7:**
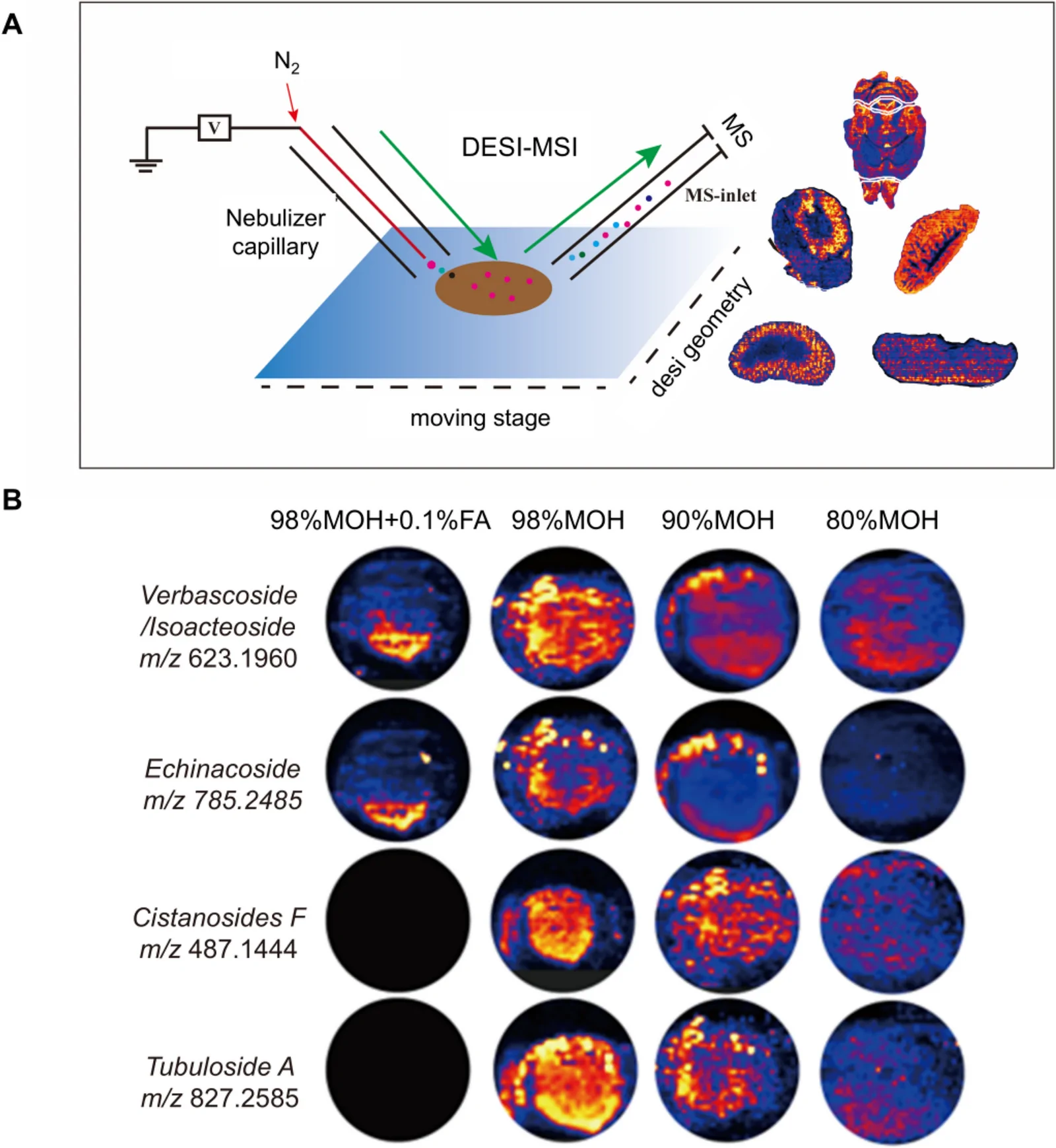
Optimization and selection of the spray solvent. **A** Schematic diagram of mass spectrometry imaging. **B** Optimization and selection of the spray solvent

To investigate the in vivo tissue-specific distribution of representative phenylethanoid glycoside constituents from CH cultivated in SAL, DESI-MSI technology was further applied for imaging analysis (Fig. 7A).

Initial optimization of imaging conditions was performed for the four previously discovered markers. However, verbascoside and isoacteoside could not be differentiated under DESI-MSI conditions, and pinoresinol yielded insufficient signal for imaging. Consequently, cistanoside F and tubuloside A, which produced stronger signals, were selected for further condition investigation. The spray solvent composition was identified as a critical factor influencing signal acquisition during imaging. Systematic optimization was therefore conducted, focusing on the effects of formic acid addition and methanol volume fraction on target signal response and image quality. Formic acid supplementation significantly suppressed the signal of target ions and reduced imaging resolution (Fig. 7B). After its exclusion, three methanol concentrations (80, 90, and 98%) were compared. Increasing methanol content progressively enhanced ionization efficiency and signal intensity, leading to the selection of 98% aqueous methanol as the optimal spray solvent for subsequent DESI-MSI analysis.

### Spatial distribution representative phenylethanoid glycosides in mouse organs

**Fig. 8:**
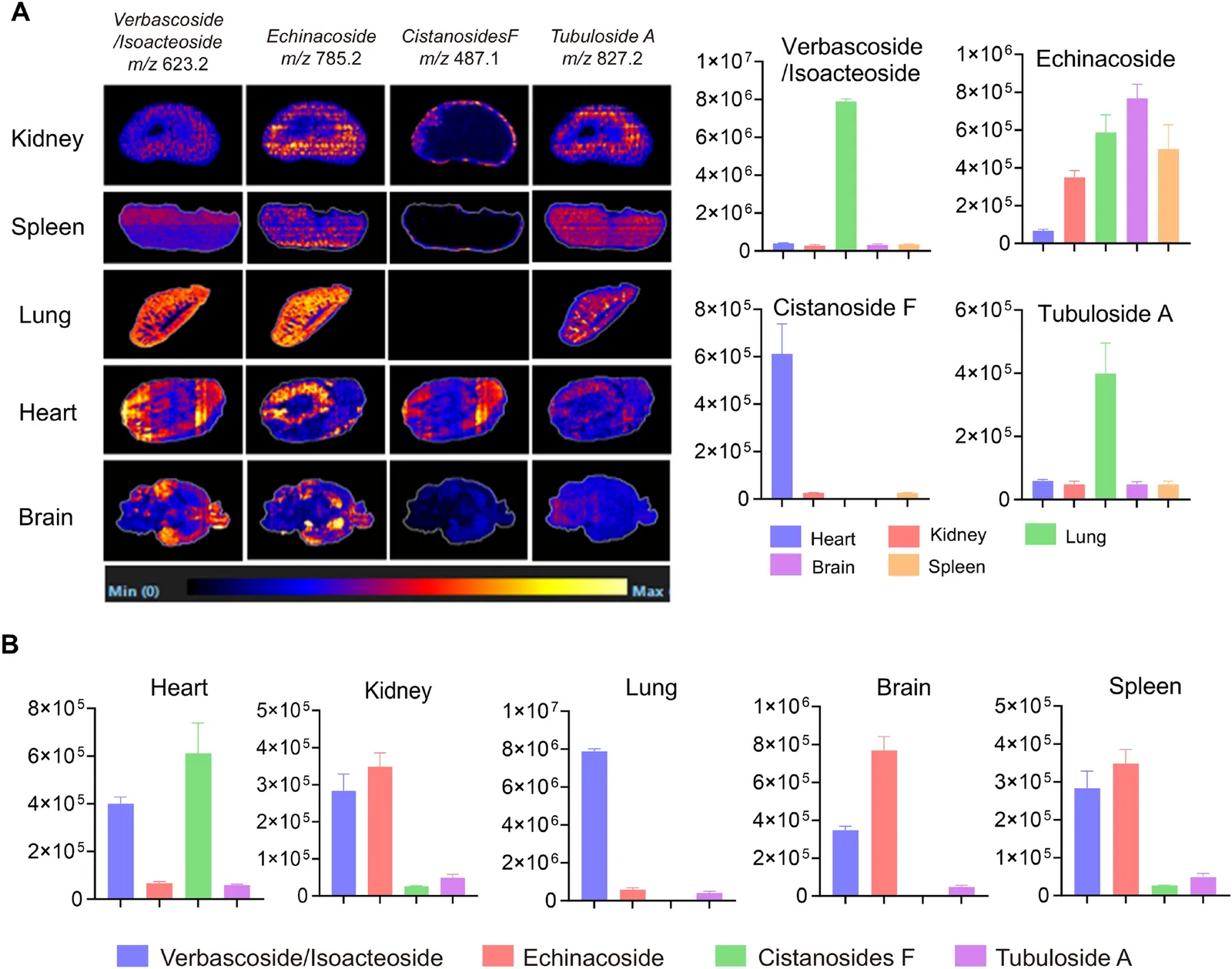
Distribution of four phenylethanoid glycosides in mouse kidney, spleen, lung, heart, and brain tissues. **A** DESI-MSI spatial imaging in mouse tissues. **B** Relative signal intensities of four phenylethanoid glycosides in mouse tissues

The optimized DESI-MSI protocol was used to visualize the in situ spatial distribution of fourresolvable constituents-verbascoside, echinacoside, isoacteoside, and pinoresinol- derived from SAL-cultivated CH, across mouse brain, heart, spleen, lung, and kidney tissues (Fig. 8). In lung tissue, verbascoside and tubuloside A were were present at markedly higher levels than in the other organs examined. Echinacoside was detectable in all organs but showed the most prominently enrichment in brain tissue, where its relative content exceeded that of the other 3 compounds. In contrast, cistanoside F exhibited specific enrichment in heart tissue and was less abundant in other tissues.

**Fig. 9:**
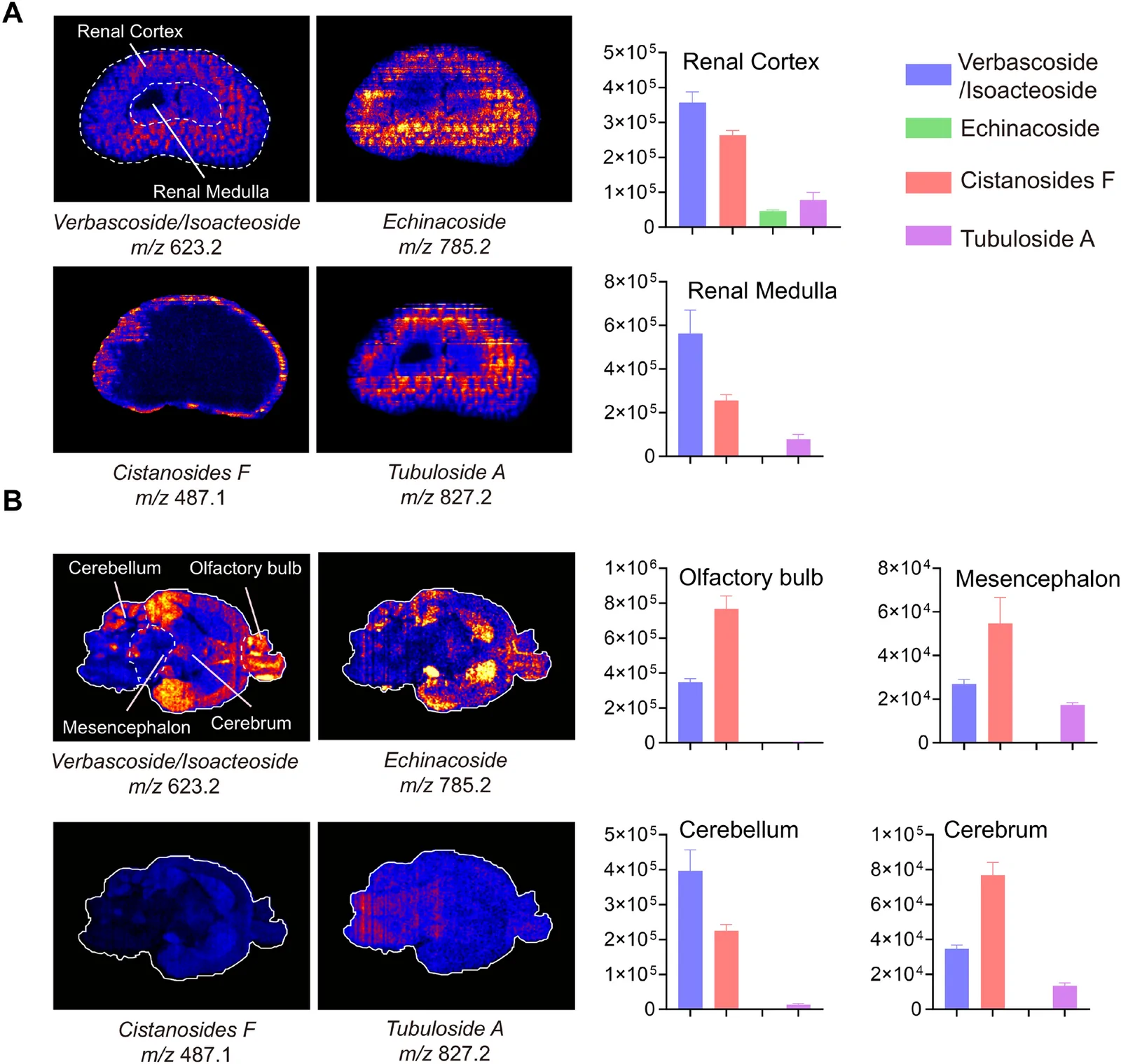
Relative enrichment of four phenylethanoid glycosides in different tissues. **A** Spatial mapping and signal intensities in renal subregions. **B** Spatial mapping and signal intensities in brain subregions

Further analysis of spatial distribution differences in kidney and brain sections (Fig. 9) revealed distinct compartment-specific patterns. In kidney tissue, verbascoside significantly more abundant in the renal medulla than in the renal cortex, whereas echinacoside and tubuloside A displayed the opposite trend. Cistanoside F was not detected in the renal medulla. In brain tissue sections, acteoside and echinacoside showed higher relative contents in the cerebrum, pons, and olfactory bulb than in the cerebellum, with echinacoside generally predominating across all brain regions. Tubuloside A was detected at relatively lower levels in brain tissues compared with the other compounds and was not detected in the olfactory bulb. No notable enrichment of cistanoside F was observed in either brain or lung tissue. These results indicated that the 4 constituents exhibit distinct tissue-specific distribution patterns in the major organs of mice.

## Discussion

This study investigated CH cultivated in the SAL of the Yellow River Delta and established a systematic hierarchical filtering strategy. The effects of saline-alkali stress on the accumulation of secondary metabolites in CH were systematically analyzed. Four characteristic chemical markers (verbascoside, echinacoside, isoacteoside and pinoresinol) were filtered out. Considering the instrumental limitations of DESI-MSI, four representative constituents with favorable imaging performance were further selected for in vivo tissue spatial distribution characterization.

The growing environment can determine the accumulation of secondary metabolites and the quality of Chinese medicinal materials. The Yellow River Delta in Shandong Province is a typical coastal SAL, with soil salinity generally ranging from 1 to 6 g/kg and a pH of 8.2–8.9, classifying it as a region with moderate to severe salinization [26]. Saline-alkali stress results from the excessive accumulation of soluble salts and alkaline substances in the soil, leading to physiological and metabolic abnormalities through multiple pathways, including impaired water uptake by plant roots, Na⁺/K⁺ imbalance, and excessive accumulation of ROS [27, 28]. When exposed to saline-alkali stress, plants can counteract adverse effects by adjusting the biosynthesis of metabolites such as polyols and sugars. In the SAL of the Yellow River Delta, CH parasitizes the roots of *Tamarix chinensis* Lour [29]. *Tamarix chinensis* Lour is a typical salt-secreting halophyte with strong tolerance to saline-alkali conditions. Studies have shown that under saline-alkali stress, *Tamarix chinensis* Lourcan actively excrete excess sodium ions via surface salt glands while simultaneously relying on the root SOS signaling pathway to regulate ion absorption and transport, thereby maintaining intracellular ion homeostasis [30]. Furthermore, *Tamarix chinensis* Lour can significantly enhance the activity of antioxidant enzymes activate secondary metabolic pathways including those for phenylpropanoids and flavonoids, and accumulate polyphenols and flavonoids [31, 32]. Changes in the secondary metabolites of *Tamarix chinensis* Lour can significantly affect the accumulation of metabolites in the parasitic CH. Our study showed that the total polyphenol, total flavonoid, and soluble sugar contents from CH cultivated in SAL were significantly higher than those in samples from traditional desert habitats, suggesting that saline-alkali stress can help CH resist oxidative damage and hyperosmotic stress by strengthening its phenolic metabolic pathways and promoting the accumulation of osmoregulatory substances [33, 34]. These physiological changes are consistent with the stress response patterns observed in most salt-tolerant medicinal plants. Furthermore, our study revealed that CH from SAL exhibited distinct differences from desert-origin samples in terms of morphological traits, powder microscopic characteristics, and overall chemical metabolic profiles, indicating that the growing habitat exerts systematic regulatory effects on the quality formation of CH, ranging from phenotypic characteristics to intrinsic metabolic levels.

In systematic phytochemical research of TCM, mining environment-specific bioactive markers from massive LC–MS datasets remains a critical bottleneck. Single untargeted metabolomics screening relying solely on VIP thresholds can only capture all statistically differential metabolites, yet this strategy cannot distinguish metabolites that merely fluctuate randomly from those intrinsically correlated with saline-alkali habitat stress, resulting in abundant false-positive candidates and excessive screening targets [35, 36]. First, UPLC-QTOF-MS untargeted metabolomics acted as the primary wide-spectrum screening layer: OPLS-DA with VIP > 1 and fold-change thresholds preliminarily filtered 30 differential metabolites to eliminate compounds without significant inter-habitat variation, which laid the basic candidate pool. However, these differential metabolites only reflected content discrepancies between SAL and SL samples, without revealing whether their accumulation was inherently coupled to saline-alkali stress adaptation. To solve this defect, WGCNA was introduced as the second core screening layer: it constructed metabolite co-expression modules and quantified metabolite-habitat correlation coefficients, further extracting 12 overlapping metabolites that were both differentially abundant and strongly positively correlated with the SAL growth environment, effectively removing metabolites with random differential expression unrelated to saline-alkali stress [37, 38]. The remaining 12 candidate metabolites demanded absolute quantitative validation and multivariate dimensionality reduction analysis to identify stable, high-abundance markers specific to SAL-grown CH, a goal unattainable through qualitative untargeted profiling alone. Therefore, UPLC-TQ-MS MRM-based targeted metabolomics was adopted as the third filtering tier to precisely quantify the absolute contents of all 12 candidates across 20 sample batches, enabling straightforward identification of metabolites with markedly higher accumulation in SAL-derived CH. Prior to principal component analysis (PCA), KMO and Bartlett’s sphericity tests were performed to verify that the dataset satisfied the prerequisites for factor analysis. Subsequent PCA dimensionality reduction extracted principal components carrying high variable loadings, from which we finalized four stable, high-contribution characteristic markers: verbascoside, echinacoside, isoacteoside, and pinoresinol. This hierarchical filtering workflow forms a convergent screening chain: broad-spectrum differential screening via untargeted metabolomics, stress-associated screening using WGCNA, absolute quantitative validation by targeted metabolomics, and final PCA-based dimensionality reduction. Each analytical step offsets the drawbacks of single-indicator screening, filtering out false-positive metabolites and ultimately yielding four robust chemical markers specific to CH cultivated in SAL [39].

Traditional Chinese medicines typically exert their therapeutic effects through a holistic mechanism involving multiple constituents and pathways. Whether active ingredients can be effectively concentrated in target organs and achieve targeted distribution within specific tissue regions is a key prerequisite for their pharmacological effects [40]. Conventional pharmacokinetic methods reflect the total amount of drug constituents in the body or in tissues and organs but cannot reveal their spatial distribution within organs. DESI-MSI can visually present the spatial distribution characteristics of active constituents within tissue micro‑regions. Moreover, it operates under label‑free, matrix‑free, and ambient‑pressure conditions, making the imaging process remarkably straightforward [41, 42]. The quality of DESI-MSI imaging is closely related to the type of spray solvent used [43]. In this study, a 98% methanol‑water solution was found to enable strong imaging signals for various constituents of CH when used as the spray solvent, laying a foundation for subsequent investigations into their spatial distribution within tissues.

DESI-MSI was employed to further investigate the spatial distribution of representative chemical constituents of CH in vivo. Due to instrumental limitations, verbascoside could not be separated from isoacteoside, and pinoresinol exhibited weak tissue imaging signals. Therefore, cistanoside F, tubuloside A, echinacoside, and verbascoside with high abundance and desirable imaging performance were selected for subsequent analysis. After parameter optimization, we systematically characterized the in vivo tissue distribution of the four phenylethanoid glycosides, all of which presented distinct tissue tropism and intra-tissue regional selectivity.

Echinacoside is a well-characterized bioactive constituent of CH and has been reported to regulate the Sirt1/FoxO pathway, exert neuroprotective effects, suppress neuroinflammation, and alleviate neurodegenerative damage [44]. In this study, echinacoside was highly enriched in brain tissues, with higher relative contents in the cerebrum and olfactory bulb than in the cerebellum, implying its capability to cross the blood–brain barrier and target central nervous system tissues. Verbascoside, a representative phenylethanoid glycoside in CH with diverse pharmacological activities [44], showed obvious compartmentalization in the kidney. Its relative abundance was significantly higher in the renal cortex than in the medulla, suggesting potential targeting of cortical structures such as glomeruli and proximal convoluted tubules. Tubuloside A, a unique phenylethanoid glycoside of CH with hepatoprotective, anti-neuroinflammatory, and antioxidant properties [45], predominantly accumulated in the renal cortex. Its highest brain distribution occurred in the olfactory bulb, indicating possible entry into the central nervous system via olfactory pathways or blood–brain barrier transport. As another unique CH-derived phenylethanoid glycoside, cistanoside F possesses antioxidant and cardiovascular protective potential [46]. It exhibited a distinctive tissue-specific distribution, with predominant enrichment in cardiac tissues and higher medullary abundance in the kidney. This preferential cardiac localization provides direct spatial evidence supporting its cardiovascular protective function. Collectively, these spatial distribution characteristics further validate the pharmacological potential of key metabolites screened by metabolomics, offering in situ histological clues for interpreting the overall efficacy of SAL-cultivated CH.

## Conclusions

This study established a hierarchical filtering strategy for mass spectrometry data and successfully identified verbascoside, echinacoside, isoacteoside, and pinoresinol as key chemical biomarkers associated with saline-alkali environments in CH. The spatial distribution results revealed that these constituents were specifically enriched in mouse tissues such as the heart, brain, and lung, providing scientific support for the quality control and resource development of CH cultivated in SAL.

## Supplementary Information

Additional file 1.

## Data Availability

All data are included in the manuscript and supplementary materials. Additional raw data are available from the corresponding author upon reasonable request.

## Abbreviations

CH: *Cistanches Herba*
SAL: Saline-alkali land
SL: Sandy land
UPLC-QTOF-MS: Ultra performance liquid chromatography-quadrupole time-of-flight mass spectrometry
UPLC-TQ-MS: Ultra performance liquid chromatography-triple quadrupole mass spectrometry
LC-HRMS: Liquid chromatography-high resolution mass spectrometry
WGCNA: Weighted gene co-expression network analysis
HPLC: High‑performance liquid chromatography
PCA: Principal component analysis
KMO: Kaiser–Meyer–Olkin
DESI-MSI: Desorption electrospray ionization mass spectrometry imaging
