# Supplementary material for: Systematic identification of characteristic chemical constituents in Cistanches Herba from saline-alkali land based on hierarchical filtering of mass spectrometry data and spatial metabolomics technology
Source: Chin Med. 2026 Oct 19;21:229. doi: 10.1186/s13020-026-01526-6 (PMC13678894; doi:10.1186/s13020-026-01526-6)
Supplement: Supplementary file 1 [file 13020_2026_1526_MOESM1_ESM.docx]

**Supplementary data**

**Table S1 Sample information of *Cistanches Herba* from saline-alkali land (SAL) in Shandong and sandy land (SL) in** **Xinjiang**

| Sample ID | Collection Site | Harvest Date |
| --- | --- | --- |
| SAL-1 | Lijin County, Shandong Province | 2021 |
| SAL-2 | Lijin County, Shandong Province | 2021 |
| SAL-3 | Lijin County, Shandong Province | 2021 |
| SAL-4 | Lijin County, Shandong Province | 2021 |
| SAL-5 | Lijin County, Shandong Province | 2022 |
| SAL-6 | Lijin County, Shandong Province | 2022 |
| SAL-7 | Lijin County, Shandong Province | 2023 |
| SAL-8 | Lijin County, Shandong Province | 2023 |
| SAL-9 | Lijin County, Shandong Province | 2023 |
| SAL-10 | Lijin County, Shandong Province | 2023 |
| SL-1 | Aral Township, Hotan, Xinjiang Uygur Autonomous Region | 2022 |
| SL-2 | Fuhai County, Altay, Xinjiang Uygur Autonomous Region | 2023 |
| SL-3 | Yutian County, Xinjiang Uygur Autonomous Region | 2022 |
| SL-4 | Minfeng County, Xinjiang Uygur Autonomous Region | 2022 |
| SL-5 | Yutian County, Xinjiang Uygur Autonomous Region | 2021 |
| SL-6 | Aral Township, Hotan, Xinjiang Uygur Autonomous Region | 2022 |
| SL-7 | Yutian County, Xinjiang Uygur Autonomous Region | 2023 |
| SL-8 | Yutian County, Xinjiang Uygur Autonomous Region | 2023 |
| SL-9 | Yutian County, Xinjiang Uygur Autonomous Region | 2024 |
| SL-10 | Yutian County, Xinjiang Uygur Autonomous Region | 2024 |

**Table S2 DNA barcoding identification results of representative *Cistanche tubulosa* samples from SAL and SL**

| Sample ID | Sampling site | NCBI Accession No. | Reference species | Sequence identity |
| --- | --- | --- | --- | --- |
| SAL-3 | Lijin County, Shandong Province | LT715533.1 | Cistanche tubulosa | 661/662(99%) |
| SAL-5 | Lijin County, Shandong Province | LT715533.1 | Cistanche tubulosa | 660/662(99%) |
| SAL-8 | Lijin County, Shandong Province | LT715533.1 | Cistanche tubulosa | 661/662(99%) |
| SL-2 | Fuhai County, Altay, Xinjiang | LT715533.1 | Cistanche tubulosa | 661/662(99%) |
| SL-3 | Yutian County, Xinjiang | LT715533.1 | Cistanche tubulosa | 661/662(99%) |
| SL-4 | Minfeng County, Xinjiang | LT715533.1 | Cistanche tubulosa | 660/662(99%) |

**Table S3 The MS data of detected compounds of by HPLC-Q-TOF-MS**

| No. | Compound Name | Retention Time  (min) | Molecular Formula | m/z | Ionization Mode | Fragment Ions | 类别 |
| --- | --- | --- | --- | --- | --- | --- | --- |
| 1 | Dihydrokaempferol | 0.83 | C_15_H_12_O_8_ | 333.0594 | [M+HCCO]- | 259.0667，125.0263 | D |
| 2 | Syringin | 0.84 | C_17_H_24_O_9_ | 371.1344 | [M-H]- | 194.0589，151.0441 | D |
| 3 | Sucrose | 0.85 | C_12_H_22_O_11_ | 341.1087 | [M-H]- | 179.0566，89.0241 | D |
| 4 | 6-Deoxycatalpol | 1.92 | C_15_H_22_O_9_ | 391.1246 | [M+HCCO]- | 183.0634 | B |
| 5 | Kankanoside B | 2.22 | C_15_H_24_O_10_ | 363.1301 | [M-H]- | 153.0560，109.0296 | B |
| 6 | Geniposidic acid | 2.76 | C_18_H_22_O_10_ | 373.1146 | [M-H]- | 211.0612，149.0611，123.0433 | B |
| 7 | Kankanoside F | 2.94 | C_28_H_40_O_17_ | 623.2199 | [M-H]- | 477.1623，315.1085 | A |
| 8 | Leonuride | 3.06 | C_15_H_24_O_9_ | 347.1343 | [M-H]- | 185.0845，127.0362 | B |
| 9 | Verbasoside | 3.08 | C_20_H_30_O_12_ | 461.167 | [M-H]- | 315.1210，123.0412 | A |
| 10 | Cistanoside B | 3.08 | C_37_H_50_O_20_ | 859.284 | [M+HCCO]- | 179.0369，161.0323 | A |
| 11 | Adoxosidic acid | 3.11 | C_16_H_24_O_10_ | 375.1291 | [M-H]- | 167.0711，123.0444 | B |
| 12 | Kankanose | 3.12 | C_27_H_38_O_18_ | 709.2183 | [M+HCCO]- | 179.0325，135.0412 | D |
| 13 | Bartsioside | 3.13 | C_15_H_22_O_8_ | 375.1302 | [M+HCCO]- | 161.0445，151.0761 | A |
| 14 | Kankanoside M | 3.14 | C_15_H_22_O_8_ | 375.1296 | [M+HCCO]- | 151.0768，133.0662 | B |
| 15 | Ferulic acid | 3.31 | C_10_H_10_O_4_ | 193.0509 | [M-H]- | 178.0261，134.0376 | D |
| 16 | Cistanoside G | 3.47 | C_20_H_30_O_11_ | 445.1738 | [M-H]- | 153.0559，119.0506 | A |
| 17 | Mussaenosidic acid | 3.52 | C_16_H_24_O_10_ | 375.1287 | [M-H]- | 213.0769，169.0892，151.0757 | B |
| 18 | Cistanoside F | 3.72 | C_21_H_28_O_13_ | 487.1469 | [M-H]- | 251.0568，161.0248，135.0454 | A |
| 19 | Caffeic acid | 3.72 | C₉H₈O₄ | 179.0347 | [M-H]- | 135.0326，93.0341 | D |
| 20 | Tubuloside C1/C2 | 3.78 | C_35_H_46_O_21_ | 801.2451 | [M-H]- | 785.2497，623.2188 | A |
| 21 | Kankanoside D | 3.94 | C_15_H_26_O_7_ | 363.1662 | [M+HCCO]- | 161.0248，135.0452 | B |
| 22 | Cistanoside H | 4.11 | C_22_H_32_O_13_ | 503.1781 | [M-H]- | 461.1672，315.1088，113.0247 | A |
| 23 | Cyanoside | 4.27 | C_29_H_34_O_15_ | 621.1829 | [M-H]- | 459.1509，179.0353，135.0453 | A |
| 24 | Echinacoside | 4.31 | C_35_H_46_O_20_ | 785.2498 | [M-H]- | 623.2105，477.1611，179.0353 | A |
| 25 | Campneoside II | 4.5 | C_29_H_36_O_16_ | 639.194 | [M-H]- | 621.1882，459.1509，179.0248，161.0248 | A |
| 26 | Cistanoside | 4.86 | C_29_H_34_O_15_ | 845.2727 | [M+HCCO]- | 179.0354，135.0452 | A |
| 27 | Purpureaside C | 4.94 | C_35_H_46_O_20_ | 785.2467 | [M-H]- | 623.2181 | A |
| 28 | Syringaresinol-4-O-β-D-glucopyranoside | 5.08 | C_28_H_36_O_13_ | 579.2086 | [M-H]- | 417.1554，181.0499 | C |
| 29 | Gluroside | 5.11 | C_15_H_24_O_8_ | 331.1393 | [M-H]- | 161.0458，151.0198 | B |
| 30 | Poliumoside | 5.33 | C_35_H_46_O_19_ | 769.2554 | [M-H]- | 623.2166，461.1667，305.0886，161.0248，145.0300 | A |
| 31 | Dehydrodiconiferyl alcohol 4-O-glucoside | 5.37 | C_26_H_32_O_11_ | 565.1919 | [M+HCCO]- | 403.1397，343.1186，145.0300，71.0136 | C |
| 32 | Cistanoside A | 5.38 | C_36_H_48_O_20_ | 799.261 | [M-H]- | 623.2094，161.0430 | A |
| 33 | Forsythoside F | 5.61 | C_34_H_44_O_19_ | 755.2402 | [M-H]- | 593.2085，161.0246 | C |
| 34 | Eucommin A | 5.66 | C_27_H_34_O_12_ | 595.2036 | [M+HCCO]- | 387.1422，181.0475 | C |
| 35 | Isocampneoside I | 5.69 | C_30_H_38_O_16_ | 653.2091 | [M-H]- | 621.1848，179.0336，161.0231，133.0284 | A |
| 36 | Eleutheroside E | 5.69 | C_34_H_46_O_18_ | 741.2577 | [M-H]- | 637.1790，381.1166 | D |
| 37 | 8-Epiloganic acid | 5.7 | C_16_H_24_O_9_ | 359.1349 | [M-H]- | 197.0815，135.0820 | B |
| 38 | Tubuloside A | 5.94 | C_37_H_48_O_21_ | 827.2606 | [M-H]- | 623.27，477.1435，161.0249 | A |
| 39 | Syringalide A 3′-α-L-rhamnopyranoside | 6.08 | C_29_H_36_O_14_ | 607.2035 | [M-H]- | 445.1642，179.0353，161.0248 | C |
| 40 | Pinoresinol 4-O-β-D-glucopyranoside | 6.18 | C_26_H_32_O_11_ | 565.1932 | [M+HCCO]- | 357.1455，151.0335 | C |
| 41 | Eutigoside A | 6.28 | C_23_H_26_O_9_ | 491.1581 | [M+HCCO]- | 163.0431，135.0453 | D |
| 42 | Kankanoside A | 6.34 | C_16_H_26_O_8_ | 345.1555 | [M-H]- | 183.1027，165.0921 | B |
| 43 | Pinoresinol | 6.36 | C_20_H_22_O_6_ | 357.1345 | [M-H]- | 151.0402，136.0569 | C |
| 44 | Kankanoside H2 | 6.52 | C_37_H_48_O_20_ | 857.2716 | [M+HCCO]- | 665.2330，623.2008，161.0248 | A |
| 45 | Isocistanoside C | 6.55 | C_30_H_38_O_15_ | 637.2127 | [M-H]- | 461.1672，161.0248，135.0456 | A |
| 46 | Isoacteoside | 6.56 | C_29_H_36_O_15_ | 623.1969 | [M-H]- | 461.1672，315.1090，179.0353，161.0247 | A |
| 47 | Verbascoside | 6.58 | C_29_H_36_O_15_ | 623.1969 | [M-H]- | 461.1672，315.1090，179.0353，161.0247 | A |
| 48 | Kankanoside H1 | 6.66 | C_37_H_48_O_20_ | 811.2666 | [M-H]- | 665.2291，623.2008，145.0297 | A |
| 49 | 2′-O-Acetylpoliumoside | 6.67 | C_37_H_48_O_20_ | 811.2664 | [M-H]- | 623.2010，481.1137，225.0769 | A |
| 50 | Campneoside | 6.68 | C_30_H_38_O_16_ | 653.2079 | [M-H]- | 491.1779，179.0353，161.0247 | A |
| 51 | Isosyringalide 3′-α-L-rhamnopyranoside | 6.81 | C_29_H_36_O_14_ | 607.2038 | [M-H]- | 461.1571，315.1023，179.0326，161.0265，135.0412 | C |
| 52 | Pheliposide | 6.95 | C_36_H_46_O_20_ | 797.2503 | [M-H]- | 635.2023，161.0247 | A |
| 53 | Cistanoside C | 7.09 | C_30_H_38_O_15_ | 637.2124 | [M-H]- | 461.1670，179.0352 | A |
| 54 | Plantainoside C | 7.09 | C_29_H_36_O_16_ | 637.2124 | [M-H]- | 161.0247，151.0399 | D |
| 55 | Caffeoylpoliumoside | 7.15 | C_44_H_52_O_20_ | 947.2838 | [M-H]- | 783.2364，161.0249 | A |
| 56 | (−)-Catechin gallate | 7.17 | C_22_H_18_O_10_ | 487.0879 | [M+HCCO]- | 179.0352 | D |
| 57 | Kankanoside I | 7.3 | C_30_H_38_O_16_ | 753.2617 | [M-H]- | 591.2298，161.0247 | A |
| 58 | 2′-Acetylverbascoside | 7.38 | C_31_H_38_O_16_ | 665.2094 | [M-H]- | 623.2086，461.1664，135.0452 | A |
| 59 | Osmanthuside B6 | 7.76 | C_29_H_36_O_13_ | 591.2084 | [M-H]- | 163.0412，145.0223 | A |
| 60 | Tubuloside B | 8.21 | C_31_H_38_O_16_ | 665.2091 | [M-H]- | 623.1994，315.1084，161.024 | A |
| 61 | Tubuloside E | 8.37 | C_31_H_38_O_15_ | 649.2143 | [M-H]- | 503.1785，461.1663，163.039 | A |
| 62 | Salsaside F | 8.37 | C_31_H_38_O_15_ | 649.2139 | [M-H]- | 607.2028，145.0297 | A |
| 63 | Kankanoside N | 8.4 | C_16_H_28_O_8_ | 347.1714 | [M-H]- | 161.0431，113.0246 | B |
| 64 | Salsaside B | 8.62 | C_28_H_34_O_13_ | 623.1988 | [M+HCCO]- | 161.0216，133.0369 | D |
| 65 | Kankanoside J1 | 8.72 | C_32_H_40_O_17_ | 695.2189 | [M-H]- | 653.2026，459.1560 | A |
| 66 | Salsaside A | 8.74 | C_28_H_34_O_13_ | 577.1903 | [M-H]- | 179.0351，161.0246 | A |
| 67 | Cistansalside E | 9.58 | C_21_H_32_O_12_ | 591.2085 | [M-H]- | 179.0348，161.0246，135.0449 | A |
| 68 | Osmanthuside B6 | 10.61 | C_29_H_36_O_13_ | 591.2086 | [M-H]- | 445.1698，145.0298 | A |
| 69 | Tubuloside C | 10.88 | C_43_H_54_O_24_ | 953.294 | [M-H]- | 749.2481，477.1574，161.0236 | A |
| 70 | Cistansalside C | 11.39 | C_27_H_40_O_13_ | 571.2392 | [M-H]- | 359.1330，161.0242 | A |
| 71 | Kankanoside C | 11.41 | C_27_H_42_O_17_ | 445.1142 | [M+HCCO]- | 145.0297 | B |
| 72 | Tubuloside D | 11.74 | C_43_H_54_O_23_ | 937.2973 | [M-H]- | 791.2583，145.0244 | A |
| 73 | Citrusin A | 11.99 | C_26_H_34_O_12_ | 537.1985 | [M-H]- | 261.0762，219.0651，107.0503 | C |
| 74 | Salsaside C | 15.18 | C_28_H_34_O_13_ | 607.2036 | [M+HCCO]- | 415.1555，269.0045 | D |

**Table S4 12 potential characteristic components of *Cistanches Herba* in saline alkali soil**

| No. | Compound Name | *m/z* | Mass Error  (ppm) | Ionization Mode |
| --- | --- | --- | --- | --- |
| 1 | Verbascoside | 623.1948 | -5.307 | [M-H]^-^ |
| 2 | Echinacoside | 785.2498 | -1.527 | [M-H]^-^ |
| 3 | Verbasoside | 357.1345 | 0.3901 | [M-H]^-^ |
| 4 | Cistanoside F | 623.2199 | 1.0759 | [M-H]^-^ |
| 5 | Purpureaside C | 785.2467 | -5.465 | [M-H]^-^ |
| 6 | Pinoresinol | 357.1345 | 0.3901 | [M-H]^-^ |
| 7 | Poliumoside | 769.2554 | -0.8588 | [M-H]^-^ |
| 8 | 8-Epiloganic acid | 359.1349 | 0.4474 | [M-H]^-^ |
| 9 | Sucrose | 341.1087 | -0.7204 | [M-H]^-^ |
| 10 | Campneoside II | 639.194 | 1.2541 | [M-H]- |
| 11 | Isoacteoside | 623.1969 | -0.249 | [M-H]- |
| 12 | Tubuloside A | 827.2606 | 2.0011 | [M-H]- |

**Table S5 Stability of analytical method**

| No. | Compound | Time Interval （h）  Content（ug / g） | | | | | | | | RSD（%） |
| --- | --- | --- | --- | --- | --- | --- | --- | --- | --- | --- |
|  |  | 0 | 1 | 2 | 4 | 6 | 8 | 12 | 24 |  |
| 1 | Verbascoside | 3780 | 3696 | 3720 | 3668 | 3824 | 3764 | 3848 | 3796 | 1.67 |
| 2 | Echinacoside | 10000 | 10080 | 9800 | 9840 | 9960 | 9840 | 9560 | 9920 | 1.59 |
| 3 | Verbasoside | 13.8 | 14.24 | 14.28 | 14.12 | 14.12 | 13.96 | 14.04 | 14 | 1.10 |
| 4 | Cistanoside F | 184 | 181.2 | 182.4 | 178.4 | 178 | 180 | 179.2 | 175.2 | 1.53 |
| 5 | Purpureaside C | 2704 | 2692 | 2644 | 2604 | 2628 | 2636 | 2620 | 2612 | 1.38 |
| 6 | Pinoresinol | 142.8 | 141.6 | 143.2 | 141.2 | 143.6 | 142.4 | 142.4 | 146.4 | 1.11 |
| 7 | Poliumoside | 10.76 | 10.28 | 10.44 | 10.76 | 10.644 | 10.56 | 10.4 | 10.44 | 1.66 |
| 8 | 8-Epiloganic acid | 37 | 37.64 | 36.92 | 37.08 | 36.92 | 37.16 | 37.08 | 37.08 | 0.61 |
| 9 | Sucrose | 4560 | 4640 | 4520 | 4680 | 4600 | 4680 | 4600 | 4720 | 1.45 |
| 10 | Campneoside II | 123.6 | 121.2 | 124.4 | 124.8 | 122.4 | 128.4 | 123.2 | 126.4 | 1.83 |
| 11 | Isoacteoside | 2996 | 3056 | 2992 | 2964 | 2952 | 2936 | 2964 | 2972 | 1.23 |
| 12 | Tubuloside A | 728 | 740 | 744 | 748 | 724 | 744 | 756 | 740 | 1.39 |

**Table S6 Accuracy and precision of analytical methods**

| No. | Compound | Nominal Concentration （ng / mL） | Measured Concentration （ng / mL） | Precision（RSD %） | Accuracy（RE %） |
| --- | --- | --- | --- | --- | --- |
| 1 | Verbascoside | 0.6 | 0.62 | 2.21 | 103.78 |
| 2 | Echinacoside | 0.6 | 0.60 | 1.05 | 100.69 |
| 3 | Verbasoside | 0.6 | 0.61 | 1.62 | 101.88 |
| 4 | Cistanoside F | 0.6 | 0.60 | 0.88 | 100.40 |
| 5 | Purpureaside C | 0.6 | 0.59 | 0.47 | 99.31 |
| 6 | Pinoresinol | 0.6 | 0.60 | 0.97 | 100.57 |
| 7 | Poliumoside | 0.6 | 0.59 | 1.37 | 99.33 |
| 8 | 8-Epiloganic acid | 0.6 | 0.58 | 1.12 | 98.08 |
| 9 | Sucrose | 0.6 | 0.61 | 1.34 | 101.73 |
| 10 | Campneoside II | 0.6 | 0.60 | 1.82 | 101.16 |
| 11 | Isoacteoside | 0.6 | 0.59 | 1.47 | 99.93 |
| 12 | Tubuloside A | 0.6 | 0.59 | 1.07 | 99.55 |

**Table S7 Accuracy and precision of analytical methods**

| No. | Compound | Repeatability (n=6) | | Recovery (n=6) | |
| --- | --- | --- | --- | --- | --- |
|  |  | Mean ± SD | RSD（%） | Recovery（%） | RSD（%） |
| 1 | Verbascoside | 3757.33 ± 29.90 | 0.79 | 97.39 | 1.02 |
| 2 | Echinacoside | 10093.33 ± 94.28 | 0.93 | 102.11 | 0.48 |
| 3 | Verbasoside | 13.97 ± 0.31 | 2.25 | 101.48 | 1.38 |
| 4 | Cistanoside F | 179.13 ± 3.65 | 2.04 | 100.84 | 0.36 |
| 5 | Purpureaside C | 2683.33 ± 48.79 | 1.81 | 100.00 | 2.53 |
| 6 | Pinoresinol | 141.80 ± 2.62 | 1.84 | 99.86 | 1.12 |
| 7 | Poliumoside | 10.22 ± 0.17 | 1.70 | 98.33 | 1.33 |
| 8 | 8-Epiloganic acid | 37.50 ± 0.26 | 0.70 | 102.48 | 1.51 |
| 9 | Sucrose | 4600.00 ± 46.18 | 1.00 | 104.40 | 1.39 |
| 10 | Campneoside II | 123.06 ± 1.56 | 1.26 | 100.58 | 1.20 |
| 11 | Isoacteoside | 3021.33 ± 53.09 | 1.75 | 100.18 | 0.89 |
| 12 | Tubuloside A | 740.00 ± 12.00 | 1.62 | 100.22 | 1.05 |

**Figure S1 Fragmentation pattern of representative phenylethanoid glycosides****Figure S2 Fragmentation pattern of representative iridoid glycosides**

**Figure S3 Fragmentation pattern of representative lignans**

**Figure S4. (A) PCA of chemical components in *Cistanches Herba* from saline-alkali land (SAL) and sandy land (SL). (B) Permutation test (n = 200) for the OPLS-DA model acquired under negative-ion mode.**

**Figure S5 Quantitative standard curves for each analyte**

**Figure S6 Structures of the 12 candidate characteristic components**
